# The composition of peripheral immunocompetent cell subpopulations and cytokine content in the brain structures of mutant Disc1-Q31L mice

**DOI:** 10.18699/VJ20.672

**Published:** 2020-11

**Authors:** M.M. Gevorgyan, S.Ya. Zhanaeva, E.L. Alperina, T.V. Lipina, G.V. Idova

**Affiliations:** Scientific Research Institute of Physiology and Basic Medicine, Novosibirsk, Russia; Scientific Research Institute of Physiology and Basic Medicine, Novosibirsk, Russia; Scientific Research Institute of Physiology and Basic Medicine, Novosibirsk, Russia; Scientific Research Institute of Physiology and Basic Medicine, Novosibirsk, Russia; Scientific Research Institute of Physiology and Basic Medicine, Novosibirsk, Russia

**Keywords:** Disc1-Q31L mice, cytokines, T cells, B cells, antibody-forming cells, brain, peripheral blood, spleen, Disc1-Q31L мыши, цитокины, Т-клетки, В-клетки, антителообразующие клетки, мозг, периферическая кровь, селезенка

## Abstract

The DISC1 (disrupted in sсhizophrenia 1) gene is associated with brain dysfunctions, which are involved in a variety of mental disorders, such as schizophrenia, depression and bipolar disorder. This is the first study to examine the immune parameters in Disc1-Q31L mice with a point mutation in the second exon of the DISC1 gene compared to mice of the C57BL/6NCrl strain (WT, wild type). A flow cytometry assay has shown that intact Disc1- Q31L mice differ from the WT strain by an increase in the percentage of CD3+ T cells, CD3+CD4+ Т helper cells and CD3+CD4+CD25+ T regulatory cells and a decrease in CD3+CD8+ T cytotoxic/suppressor cells in the peripheral blood. A multiplex analysis revealed differences in the content of cytokines in the brain structures of Disc1-Q31L mice compared to WT mice. The content of pro-inflammatory cytokines was increased in the frontal cortex (IL-6, IL- 17 and IFNγ) and striatum (IFNγ), and decreased in the hippocampus and hypothalamus. At the same time, the levels of IL-1β were decreased in all structures being examined. In addition, the content of anti-inflammatory cytokines IL-4 was increased in the frontal cortex, while IL-10 amount was decreased in the hippocampus. Immune response to sheep red blood cells analyzed by the number of antibody-forming cells in the spleen was higher in Disc1-Q31L mice at the peak of the reaction than in WT mice. Thus, Disc1-Q31L mice are characterized by changes in the pattern of cytokines in the brain structures, an amplification of the peripheral T-cell link with an increase in the content of the subpopulations of CD3+CD4+ T helpers and CD3+CD4+CD25+ T regulatory cells, as well as elevated immune reactivity to antigen in the spleen.

## Abstract (ru)

Нарушения в гене DISC1 (disrupted in sсhizophrenia 1) ассоциированы с дисфункциями мозга, ха- рактерными для ряда психических заболеваний (шизофрения, депрессия, биполярное расстройство и др.). В данной работе впервые изучены иммунологические параметры у мышей линии Disc1-Q31L с точечной му- тацией во втором экзоне гена DISC1 (замена глутамина на лейцин в 31-м положении) по сравнению с мыша- ми линии C57BL/6NCrl (дикий тип). Методом проточной цитофлуориметрии показано, что по сравнению с мышами дикого типа у интактных Disc1-Q31L мышей в периферической крови увеличено процентное содер- жание CD3+ Т-лимфоцитов, CD3+CD4+ Т-хелперов и CD3+CD4+CD25+ Т-регуляторных клеток при снижении CD3+CD8+ Т-цитотоксических/супрессорных клеток. С помощью мультиплексного анализа выявлены разли- чия в содержании цитокинов в структурах мозга Disc1-Q31L мышей по сравнению с мышами дикого типа. Содержание провоспалительных цитокинов повышалось во фронтальной коре (IL-6, IL-17 и IFNγ) и стриатуме (IFNγ), а в гиппокампе и гипоталамусе, напротив, уменьшалось. При этом IL-1β снижался во всех исследован- ных структурах. Наряду с этим обнаружено увеличение количества противовоспалительного цитокина IL-4 во фронтальной коре и снижение IL-10 в гиппокампе. Иммунная реактивность на введение антигена эритро- цитов барана, анализируемая по числу антителообразующих клеток в селезенке, на пике иммунного ответа у Disc1-Q31L мышей была выше, чем у мышей дикого типа. Таким образом, мыши линии Disc1-Q31L характе- ризуются изменением паттерна цитокинов в структурах мозга, усилением периферического Т-клеточного звена с повышением субпопуляций CD3+CD4+ Т-хелперов и CD3+CD4+CD25+ Т-регуляторных клеток, а также увеличением иммунной реактивности на антиген в селезенке.

## Introduction

It is now well established that a variety of social, environmental and genetic factors may cause inflammatory responses that, over time, may result in development of multiple diseases, including neuropsychiatric disorders (Haroon et al., 2012; Felger, Lotrich, 2013; Dantzer, 2018). The inflammatory processes are closely associated with alterations in the production of cytokines (IL-6, IL-2, IL-1β, TNFα, etc.), the composition of T-cell subsets with different functional activities (CD4+ T- helper cells, CD8^+^ cytotoxic/suppressor T-cells, T-regulatory cells), both in the peripheral immune system and in the central nervous system (Haroon et al., 2012; Felger, Lotrich, 2013; Dantzer, 2018). Animal models have provided valuable opportunities to study the impact of immune dysfunctions and related alterations in neurotransmitter and hormonal systems in the pathogenesis of neuropsychiatric disorders caused by multiple risk factors, including genetic background. As shown previously, animals with genetic predisposition to depressive or aggressive behavior are characterized by changes in the distribution and ratio of the main subpopulations of T-cells in the blood and spleen, immune responsiveness to T-dependent antigen, as well as cytokine variations in the periphery and brain structures (Alperina et al., 2007, 2019; Idova et al., 2013, 2015, 2019; Takahashi et al., 2018).

Disrupted-in-Schizophrenia-1 (DISC1) gene has been functionally linked to brain dysfunctions associated with impaired neurodevelopment processes and intracellular signaling pathways that predispose to schizophrenia, major depression, and bipolar disorder (Lipina et al., 2010; Hikida et al., 2012; Mathieson et al., 2012; Lipina, Roder, 2014; Serykh еt al., 2020). Several mouse models based on DISC1 dysfunction have been generated to date, including a homozygous Disc1-Q31L^–/–^ mouse line with a point mutation in exon 2 of chromosome 8, leading to glutamine to leucine substitution at amino acid 31 in the DISC1 protein (Q31L). Analysis of emotional, social and cognitive behaviors of this mice line showed a range of behavioral abnormalities that may be considered as a depression-like endophenotype (Lipina et al., 2013; Lipina, Roder, 2014; Dubrovina et al., 2018; Serykh еt al., 2020). The Q31L mutation in DISC1 gene is also known to be associated with changes in the dopaminergic (DA) activity (Lipina et al., 2013) and other neuromediator systems, which are involved in the neurobiological mechanisms of psychiatric disorders and in the control of immune function (Saurer et al., 2006; Devoino et al., 2009; Al’perina, 2014).

However, peculiar changes in immunological variables in the peripheral immune system and in the brain characteristic of Disc1-Q31L^–/–^ mice remain to be elucidated. Given a role of the immune system both in the development of different psychoemotional states and in neuroimmunomodulation (Devoino et al., 2009; Idova et al., 2018, 2019; Alperina et al.,

2019), the aim of this study was to analyze the basal content of T- and B-cells in the peripheral blood and spleen, as well as the level of pro- and anti-inflammatory cytokines in the brain structures of Disc1-Q31L^–/–^ mice. Immune reactivity to the antigen by the number of antibody-forming cells (AFC) was also determined.

## Materials and methods

**Animals.** The experiments were performed in adult (3.0– 3.5 months old) homozygous male mice of the Disc1-Q31L^–/–^ strain (n = 23) and there wild type (WT) littermates of the C57BL/6NCrl strain (n = 23) weighing 27–30 g. Mice were bred in the animals facility of the Scientific Research Institute of Physiology and Basic Medicine (“Biological collection – genetic biomodels of neuropsychic diseases”, No. 493387). Mice were kept in standard cages (OptiMice Biotech A.S.; 40 × 25 × 15 cm ) in groups for 5 animals per cage under standard vivarium conditions and free access to food and water. All experimental procedures were performed in accordance with the requirements of the European Community Directive (86/609/EC) and approved by Local Ethical Committee of the Scientific Research Institute of Physiology and Basic Medicine, protocol No. 10 (17.12.2015).

**Design of experiments.** The levels of T- and B-lymphocytes and their subpopulations in the peripheral blood and spleen, as well as the content of proinflammatory (IL-1β, IL-2, IL-6, IL-17, TNFα, IFNγ) and anti-inflammatory (IL-4 and IL-10) cytokines in the brain structures (prefrontal cortex, striatum, hippocampus, hypothalamus) were assessed in intact mice of the Disc1-Q31L and WT strains (10 animals of each strain). The immune reactivity to sheep red blood cells (SRBC) was analyzed by measuring the number of antibody-forming cells (AFC) in the spleen of mice of both strains (n = 13 of each strain). SRBC were suspended in saline and injected once, intravenously into the tail vein at a dose of 5 · 10^8^.

Blood was immediately collected after the animals were decapitated into tubes containing K3EDTA (Becton Dickinson, USA). Spleens were removed on ice on day 4 after SRBC injection and placed in tubes with cooled RPMI-1640 medium (Sigma-Aldrich, USA). Brain structures were dissected on ice; brain samples were frozen in liquid nitrogen and stored at –70 °C until analysis.

**Determination of cell subpopulation.** To analyze cell subsets, 25 μl of blood was incubated for 30 minutes in a dark place with 1.5 μl (0.2–0.5 μg/μl) labeled rat anti-mouse monoclonal antibodies (MoAB) against surface markers: СD3 (allophycocyanin, APC), СD4 (peridinin-chlorophyll protein, perСP), СD8 (phycoerythrin, PE), СD25 (Brilliant Violet 421), СD19 (fluorescein isothiocynate, FITС) (all obtained from BD Pharmingen™, USA). Erythrocytes of the blood were lysed with Lysing Solution BD FASC (Becton Dickinson, USA). After a 10-minute incubation, the cells were washed once with phosphate buffered saline (PBS), the cell pellet was resuspended in 100 μl of PBS.

The spleen was cut into several pieces, and then disaggregated mechanically into single-cell suspension, which was passed through a 50 μm cell strainer. The suspension was washed twice with RPMI-1640 medium at 200 g for 5 minutes. The cell pellet was resuspended in RPMI-1640 medium, adjusted to 1 · 10^6^/100 μl of the suspension and placed into plates in a volume of 100 μl in each well. The cell suspension was incubated with the same MoAB as the blood cells for 20 minutes, and fixed by adding 1 % paraformaldehyde to each tube. Isotypic antibodies were used as a control.

The study of cell populations was performed on a FACS CANTO™ II flow cytometer (Becton Dickinson, USA) using multi-stage gating. At least 50 000 cells were analyzed in each sample. Data analysis was performed using the FACSDiva software. The contents of CD3^+^ T-lymphocytes, CD3+CD4+ T-helpers, CD3^+^CD8^+^ cytotoxic/suppressor T-lymphocytes, CD3^+^CD4^+^CD25^+^ T-regulatory cells, CD19^+^ B-lymphocytes as a percentage of the total number of cells were determined. Immunoregulatory index was measured as a ratio of the content of CD4^+^ to CD8^+^ Т-cells.

**Determination of cytokines in the brain structures.** For the analysis of cytokines, detergent-soluble fractions of brain tissues were prepared. The samples were thawed on ice, homogenized in lysis buffer cooled to +4 °C containing PBS (pH 7.4), 0.1 % Triton X-100, 1 mM EDTA, and 1 mM PMSF using plastic pestles. The homogenates were incubated on ice for 30–40 minutes. The tissue extracts were centrifuged (Centrifuge 5415 R) at 4500 rpm for 20 minutes at +4 °C. Cytokine concentrations were determined in the supernatants. The concentration was normalized to tissue weight (pg/g tissue)

The content of cytokines in brain homogenates was determined according to the manufacturer’s protocol by multiplex immunoassay on a multiplex protein and nucleic acid analyzer (Milliplex Luminex 200, Merk Millipore) using a kit (Milliplex MAP Mouse Cytokine/Chemokine, Millipore). The results were analyzed using the xPONENT and Analist software.

**Determination of antibody-forming cells.** The immune response was assessed by the relative (per 106 spleen cells) and absolute (per total number of cells in the spleen) number of IgM-AFC using the standard method (Ladics, 2007).

**Statistical analysis.** The data were analyzed using Statistica 10.0 software. To verify whether data were normally distributed, the Kolmogorov–Smirnov and Shapiro–Wilk tests were used. Normally distributed data (the content of T-cells and their subpopulations, and B-cells) were assessed by one-way ANOVA. The Mann–Whitney test was used for abnormally distributed data (cytokine content and AFC number). Data are presented as mean and mean error (M ± m) with significance set at a level of p < 0.05.

## Results

**Content of T-cells, their subpopulations, and B-cells in the peripheral blood and spleen of Disc1-Q31L mice.** There were differences in the content of all analyzed immunocompetent blood cells between nonimmunized Disc1-Q31L and WT mice. The percentage of CD3^+^ T-lymphocytes in mice of the Disc1-Q31L strain was significantly higher than in WT mice (F(1.18) = 45.2, p < 0.001). Analysis of T-lymphocyte subpopulations showed an increase in the content of CD3^+^CD4^+^ Т-helpers (F(1.17) = 15.5, p < 0.01) in Disc1-Q31L mice compared to WT strain, while the number of CD3+CD8+ T-cytotoxic/suppressor cells was decreased (F(1.17) = 12.6, p < 0.01). As a result, the immunoregulatory index, determined as the ratio of the content of CD4^+^ to CD8^+^ T-lymphocytes, in mutant mice was 1.3 times higher (F(1.18) = 27.5, p < 0.01) than in WT mice. The content of T-regulatory cells with the CD3^+^CD4^+^CD25^+^ phenotype in Disc1-Q31L mice was also higher than in WT mice (F(1.17) = 5.3, p < 0.05). The number of CD19^+^ B-lymphocytes was decreased in the peripheral blood of mutant mice compared to WT mice (F(1.17) = 5.7, p < 0.05) (see the Table).

**Table 1.**
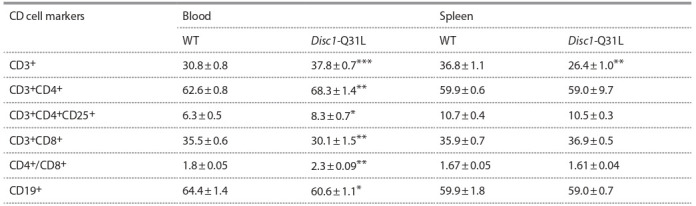
Content of the subpopulations of T- and B-lymphocytes (%) in the peripheral blood and spleen of Disc1-Q31L mice (M ± m) * p < 0.05, ** p < 0.01, *** p < 0.001 compared with WT mice (ANOVA test). Number of animals in each group = 9–10.

**Fig. 1.**
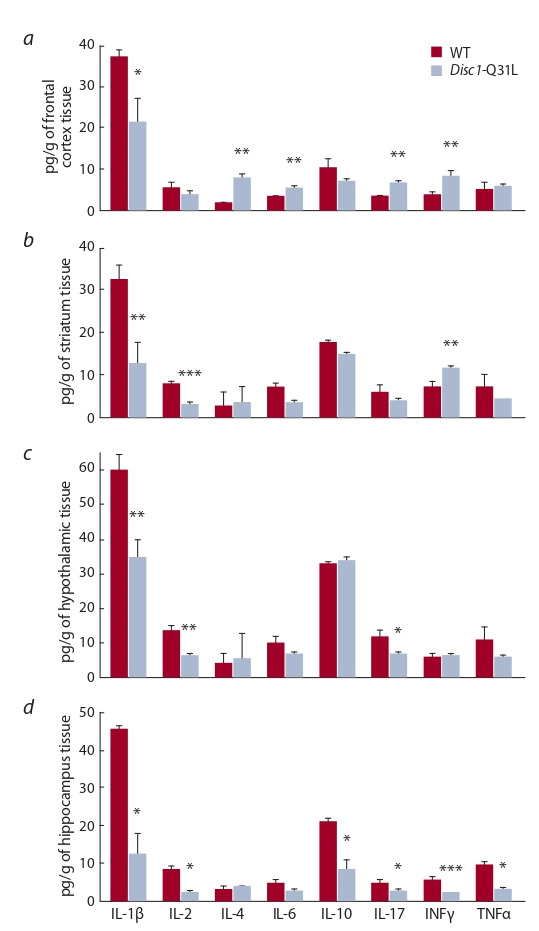
Content of cytokines in the brain structures: frontal cortex (a), striatum (b), hypothalamus (c), hippocampus (d ) in WT mice and Disc1- Q31L mice. * p < 0.05, ** p < 0.01, *** p < 0.001 compared to WT mice (Mann–Whitney U- test). Number of animals in the groups = 9–10.

**Cytokines in the brain structures in mice of the Disc1- Q31L strain.** Analysis of the cytokine profile in brain structures of intact Disc1-Q31L mice revealed regional differences in the content of cytokines between mutant and WT mice (Fig. 1).

In the frontal cortex, levels of the three pro-inflammatory cytokines IL-6 ( p < 0.01), IL-17 ( p < 0.01) and IFNγ ( p < 0.01) were higher in Disc1-Q31L mice than in WT mice, while the level of IL-1β ( p < 0.05) decreased. IL-2 and TNFα levels were similar between the mutant and WT strains ( p < 0.05). As to the content of anti-inflammatory cytokines, the level of IL-4 in Disc1-Q31L mice was higher than in WT mice ( p < 0.01), while the level of IL-10 did not change ( p > 0.05) (see Fig. 1, a).

In the striatum, the content of IFNγ ( p < 0.01) was found to be increased in Disc1-Q31L mice compared to WT animals. The levels of other pro-inflammatory cytokines – IL-1β ( p < 0.01), IL-2 ( p < 0.001) in the mutant mice were lower than in WT mice, while the levels of IL-6, IL-17 and TNFα remained unchanged ( p > 0.05). Similarly, there were no significant strain differences in the levels of anti-inflammatory cytokines IL-4 and IL-10 ( p > 0.05) (see Fig. 1, b).

When compared to WT mice, Disc1-Q31L mice showed lower levels of IL-1β ( p < 0.01), IL-2 (p < 0.01) and IL-17 ( p < 0.05) in the hypothalamus, while the levels of the rest cytokines were unchanged (IL-4, IL-6, IL-10, IFNγ, TNFα) ( p > 0.05) (see Fig. 1, c).

The levels of proinflammatory cytokines IL-1β, IL-2, IL-17, TNFα ( p < 0.05) were significantly lower in the hippocampus of Disc1-Q31L mice than in WT mice, with more pronounced decrease in IFNγ content ( p < 0.001). The levels of IL-6 were equivalent between Disc1-Q31 and WT mice ( p > 0.05). The content of anti-inflammatory cytokine IL-10 in the hippocampus of Disc1-Q31 mice was also decreased ( p < 0.05), while the level of IL-4 did not differ from that of WT mice ( p > 0.05) (see Fig. 1, d ).

**Fig. 2.**
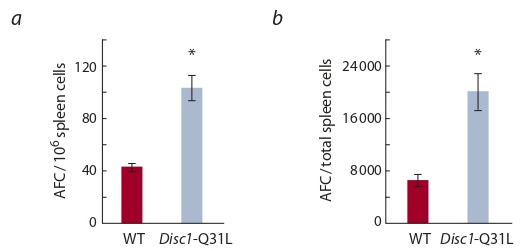
Relative (a) and absolute (b) numbers of AFC in the spleen of WT and Disc1-Q31L mice on the 4th day following immunization with SRBC (5 · 108). * p < 0.001 compared to WT mice (Mann–Whitney U-test). Number of animals in the groups = 13.

**Immune reaction of Disc1-Q31L mice to the antigen.** Immunization of Disc1-Q31L mice with SRBC produced an increase of the immune response at the peak of its development in the spleen of WT mice. The relative ( p < 0.001) and absolute ( p < 0.001) numbers of AFC in Disc1-Q31L mice were significantly higher than in WT mice (Fig. 2).

## Discussion

Changes in DISC1 protein activities caused by mutations in the DISC1 gene are known to be involved in multiple mental disorders, such as schizophrenia, depression, bipolar disorder (Lipina et al., 2010, 2013, 2014; Hikida et al., 2012; Mathieson et al., 2012). Alterations in immune variables associated with these disorders may differentially contribute to disease development. Schizophrenia has been found to be accompanied by elevated serum numbers of B-cells, along with a decrease in the content of T-cells, CD4^+^ T-helpers, and the ratio of CD4^+^ to CD8^+^ T-cells (Steiner et al., 2010). Aggressive behavior, as observed in a variety of animal models, is also associated with an increase in T-helpers and the CD4^+^/CD8^+^ ratio, as well as a higher immune response generated by an antigen (Devoino et al., 2009; Idova et al., 2015; Takahashi et al., 2018).

On the other hand, depression is characterized by increasing numbers of CD3^+^CD8^+^ T-suppressor/cytotoxic cells, a decrease in the immunoregulatory index and the immune response suppression (Alperina et al., 2007; Devoino et al., 2009; Haroon et al., 2012; Felger, Lotrich, 2013; Idova et al., 2013).

The present study demonstrates that, compared to WT mice, intact mice of the Disc1-Q31L strain have raised blood levels of CD3^+^ T-lymphocytes, and their subpopulations, such as CD3+CD4^+^ T-helpers and CD3^+^CD4^+^CD25^+^ T-regulatory cells, with a consequent increase of the immunoregulatory index. At the same time, the mutant mice showed lower percentage of CD3^+^ T-lymphocytes in the spleen, that might lead to a predominance of CD19^+^ B-cells, thereby suggesting a redistribution of these cell subsets within the immune system.

Redistribution of T- and B-lymphocytes, which are known to produce specific sets of cytokines, and the ratio of these cells in the immunocompetent organs may significantly affect inflammatory and immune processes characteristic of genetically determined behaviors and psychopathology (Ottaway, Husband, 1994; Devoino et al., 2009). It seems, thus, possible that higher ability of Disc1-Q31L mice to respond to antigen challenge, as measured by the numbers of AFC in the spleen, may be related to changes in immune cell distribution among different compartments of the immune system.

Our results have also shown that the pattern of cytokine variations over brain structures differ in Disc1-Q31L and WT mice and depends on the brain area, in which these cytokines are localized. The levels of IL-6, IL-17 and IFNγ were found to be simultaneously increased only in the frontal cortex of Disc1-Q31L mice compared to WT animals. These pro-inflammatory cytokines has long been known as very potent signaling molecules of neuroinflammation implicated in the pathophysiology of depression, bipolar disorder, and schizophrenia (Grigor’ian et al., 2014; Lesh et al., 2018). Moreover, the frontal cortex has also been associated with the development of various psychiatric diseases (Clapcote et al., 2007).

Only the IFNγ level was increased in the striatum of Disc1- Q31L mice compared to WT mice, whereas the concentrations of other cytokines decreased. Levels of pro-inflammatory cytokines were also lower in the hippocampus and hypothalamus of mutant mice than in WT mice. Changes in the distribution of brain cytokines found in Disc1-Q31L mice suggest that this mutation may contribute to neuroinflammation, which is an important etiological factor for affective disorders. The observed increase in the level of anti-inflammatory cytokine IL-4 in the frontal cortex of Disc1-Q31L mice could reflect a compensatory response to the elevations of pro-inflammatory cytokines that occurred in this brain area. These findings are consistent with previous reports, showing that various forms of depression-like behavior or aggression are associated with impaired balance between pro- and anti-inflammatory cytokines in a number of brain regions including the frontal cortex and hippocampus (Takahashi et al., 2018; Alperina et al., 2019; Idova et al., 2019).

DISC1 has been found to form a complex with other intranuclear transcription factors, which mediate the expression of several genes implicated in behavioral changes resembling human psychiatric disorders (Lipina, Roder, 2014). A wide range of studies on behavioral phenotype of Disc1-Q31L produced conflicting results. Some data indicate that mice with Q31L mutation in Disc1 have a depressive-like enodophenotype (Lipina et al., 2013; Dubrovina et al., 2018; Serykh еt al., 2020), while others did not show significant behavioral differences in this strain compared with the WT control in any of the tests (Shoji et al., 2012). Recent evidence suggests that Disc1- Q31L mice may also display aggressive behavior (Serykh еt al., 2020). In contrast to the data obtained in other models, in which animals developing depression-like responses showed immunosuppression (Alperina et al., 2007; Devoino et al., 2009; Idova et al., 2013), our results revealed higher immune reactivity in Disc1-Q31L mice compared to WT control. At the same time, immune parameters characteristic of Disc1- Q31L mice are more relevant to those observed in aggressive animals. It may be due to the diverse behavioral phenotype of these mice displaying not only depression, but also aggressive behavior (Serykh et al., 2020) that is associated with increased immune function and specific pattern of cytokines.

However, the mechanisms underlying alterations in peripheral immune parameters and the profile of brain cytokines are unknown. There is growing evidence that immune mediators such as cytokines are involved in the interactions between the immune and neuroendocrine systems and can change the activity of central neuromediator systems that contribute to cognitive, behavioral, and brain structure abnormalities seen in affective disorders (Grigor’ian et al., 2014; Lesh et al., 2018). It is possible that the immune status of Disc1-Q31L mice could be related to the neurochemical pattern of the brain characteristic of this strain. Disc1-Q31L mice have been shown to have decreased levels of DA combined with DOPAC increase in the nucleus accumbens (Lipina et al., 2013), known to implicate in neuroimmunomodulation (Saurer et al., 2006; Devoino et al., 2009; Al’perina, 2014). There is also data that the DOPAC/DA ratio, which may reflect the metabolic rate of DA and synaptic activity, increases under immunostimulation observed in animals experienced excessive aggression associated with elevated activity of the DA system (Devoino et al., 2009; Al’perina, 2014). Taking into account changing DAergic activity in the brain structures of Disc1-Q31 mice (Lipina et al., 2013) and the critical role of this system in the control of aggression and neuroimmunomodulation (Saurer et al., 2006; Devoino et al., 2009; Al’perina, 2014), it is likely that DA may contribute to the enhancement of immune function found in the Disc1-Q31 strain.

However, it remains unclear whether variations of central cytokines are related to brain alterations of monoamines specific for Disc1-Q31L mice or the Q31L mutation determines their profile. Moreover, it has been found that not only neurotransmitters can affect the production of cytokines (Kawano et al., 2018), but also cytokines can modulate mediator neurotransmission and promote changes in the neurochemical pattern of the brain (Dunn, 2006; Felger, Lotrich, 2013).

## Conclusion

Our data indicate that the Q31L point mutation in the DISC1 gene leading to the substitution of glutamine to leucine at amino acid 31 has a significant influence on immunity and may result in an amplification of peripheral T-cell link with an increase in the content of CD3+CD4+ T-helpers and CD3^+^CD4^+^CD25^+^ T-regulatory cell subpopulations, as well as elevated immune reactivity in the spleen induced by the antigen. Alterations in the peripheral immune variables are accompanied with changes in the distribution of pro- and anti-inflammatory cytokines within brain structures, which are involved both in the control of different forms of behavior and in immune function. The Disc1-Q31L mouse strain is a promising model for further study of the relationships between genetic factors and neuroimmunological mechanisms and their implication in the development of psychoemotional disorders.

## Conflict of interest

The authors declare no conflict of interest.
